# Exogenous Ghrelin Accelerates the Healing of Acetic Acid-Induced Colitis in Rats

**DOI:** 10.3390/ijms17091455

**Published:** 2016-09-01

**Authors:** Aleksandra Matuszyk, Piotr Ceranowicz, Zygmunt Warzecha, Jakub Cieszkowski, Dagmara Ceranowicz, Krystyna Gałązka, Joanna Bonior, Jolanta Jaworek, Krzysztof Bartuś, Krzysztof Gil, Rafał Olszanecki, Artur Dembiński

**Affiliations:** 1 Department of Physiology, Faculty of Medicine, Jagiellonian University Medical College, 16 Grzegórzecka St., 31-531 Cracow, Poland; aleksandra.matuszyk@uj.edu.pl (A.M.); mpwarzec@cyf-kr.edu.pl (Z.W.); jakub.cieszkowski@uj.edu.pl (J.C.); dagac@op.pl (D.C.); mpdembin@cyf-kr.edu.pl (A.D.); 2 Department of Anatomy, Faculty of Medicine, Jagiellonian University Medical College, 31-034 Cracow, Poland; 3 Department of Pediatrics, Gastroenterology and Nutrition, University Children’s Hospital, Faculty of Medicine, Jagiellonian University Medical College, 30-663 Cracow, Poland; 4 Department of Pathomorphology, Faculty of Medicine, Jagiellonian University Medical College, 31-531 Cracow, Poland; krystyna.galazka@uj.edu.pl; 5 Department of Medical Physiology Faculty of Health Sciences, Jagiellonian University Medical College, 31-126 Cracow, Poland; joanna.bonior@uj.edu.pl (J.B.); jolanta.jaworek@uj.edu.pl (J.J.); 6 Department of Cardiovascular Surgery and Transplantology, Jagiellonian University, JP II Hospital, 31-202 Cracow, Poland; krzysztof.bartus@uj.edu.pl; 7 Department of Pathophysiology, Faculty of Medicine, Jagiellonian University Medical College, 31-121 Cracow, Poland; krzysztof.m.gil@uj.edu.pl; 8 Department of Pharmacology, Faculty of Medicine, Jagiellonian University Medical College, 31-531 Cracow, Poland; rafal.olszanecki@uj.edu.pl

**Keywords:** colitis, DNA synthesis, mucosal blood flow, myeloperoxidase, interleukin-1β

## Abstract

Previous studies have shown that ghrelin reduces colonic inflammation induced by trinitrobenzene sulfonic acid and dextran sodium sulfate. In the present study we determined the effect of treatment with ghrelin on the course of acetic acid-induced colitis in rats. Rectal administration of 3% acetic acid solution led to induction of colitis in all animals. Damage of the colonic wall was accompanied by an increase in mucosal concentration of pro-inflammatory interleukin-1β (IL-1β) and tumor necrosis factor-α (TNF-α), as well mucosal activity of myeloperoxidase. Moreover, induction of colitis led to a reduction in colonic blood flow and DNA synthesis. Administration of ghrelin after induction of colitis led to faster regeneration of the colonic wall and reduction in colonic levels of IL-1β, TNF-α, and myeloperoxidase. In addition, treatment with ghrelin improved mucosal DNA synthesis and blood flow. Our study disclosed that ghrelin exhibits a strong anti-inflammatory and healing effect in acetic acid-induced colitis. Our current observation in association with previous findings that ghrelin exhibits curative effect in trinitrobenzene sulfonic acid- and dextran sodium sulfate-induced colitis suggest that therapeutic effect of ghrelin in the colon is universal and independent of the primary cause of colitis.

## 1. Introduction

Ghrelin, an acylated 28-amino acid polypeptide, was first discovered in the rat and human stomach [1,2,3]. The stomach is also the main source of endogenous ghrelin [2]. Ghrelin is a natural ligand for growth hormone secretagogue receptor—GHS-R. This receptor is present mainly in the pituitary gland and hypothalamus, but in lower amounts it also occurs in other central and peripheral tissues [4]. Acting on GHS-R in the anterior lobe of the pituitary gland, ghrelin strongly and dose-dependently stimulates secretion of growth hormone [1]. In addition to the release of growth hormone, ghrelin has other biological effects, such as stimulation of gastrointestinal motility [5] and food intake [6].

Previous experimental studies have shown that ghrelin exhibits a protective effect in several organs, including the heart [7], kidney [8], spinal cord [9], and brain against ischemia-induced damage [10], as well as reduces the sepsis-induced acute lung injury and mortality in rats [11]. In the gastrointestinal tract, pretreatment with ghrelin inhibits the development of gastric ulcers induced by ethanol [12], stress [13], and alendronate [14]. Administration of ghrelin has been also shown to accelerate the healing of gastric ulcers evoked by acetic acid [15] and ethanol [16], as well as duodenal ulcers evoked by acetic acid [15] and cysteamine [17]. Protective effect of ghrelin has been also found in the pancreas [18]. Pretreatment with this peptide inhibits the development of different type of experimental pancreatitis [19,20,21] and accelerates recovery in this disease [22,23,24]. In addition, ghrelin has a therapeutic effect in the healing of oral ulcers [25] and administration of ghrelin attenuates the development of liver fibrosis in murine models of this disease [26]. The antifibrotic and anti-inflammatory effect of ghrelin in the liver seems to be related to inhibition of TGF-β1 and NF-κB signaling pathways, as well as suppression of autophagy [26].

The role of ghrelin in inflammatory bowel diseases is not clear. Clinical studies have shown that patients in the acute phase of Crohn’s disease and ulcerative colitis have higher circulating levels of ghrelin than healthy individuals [27,28,29]. Additionally, expression of ghrelin in the mucosa of the large intestine is increased in those patients [30,31]. Experimental studies performed in rats [30] and mice [32] have shown that administration of ghrelin exhibits therapeutic effect in colitis induced by trinitrobenzene sulfonic acid (TNBS). In harmony with this observation is our previous finding that administration ghrelin before enema with acetic acid reduces the severity of acetic acid-induced colitis in rats [33]. On the other hand, the research conducted by De Smet et al. [34] has shown that endogenous and exogenous ghrelin worsens the course of dextran sodium sulfate (DDS)-induced colitis in mice.

Inflammatory bowel disease (IBD) is a chronic, remitting inflammatory disorder occurring in two forms as Crohn’s disease and ulcerative colitis. There are numerous methods of IBD treatment, but there is no method that gives a permanent therapeutic effect this disease [35]. Clinical observations provide some information regarding the etiology and pathogenesis, and the course of IB. However, research on the initial events of IBD, or research on the new therapeutic methods in this disease cannot be carried out on humans for ethical reasons. Animal models of colitis are useful in solving this problem. A model of colitis evoked by enema with acetic acid in rodents is commonly used and easy to induce. This model of colitis exhibits close resemblance to clinical IBD in terms of pathogenesis of inflammation, proinflammatory cytokines, and morphological features [36,37]. In addition, acetic acid-induced colitis is a perfect model for the investigation of factors that can stimulate the regeneration of the colon. For this reason we have used this model of colitis in our current study.

The aim of this study was to determine whether administration of ghrelin after the development of acetic acid-induced colitis in rats, affects the course of this inflammation.

## 2. Results

### 2.1. Morphological Signs of Colitis

**Figure 1:**
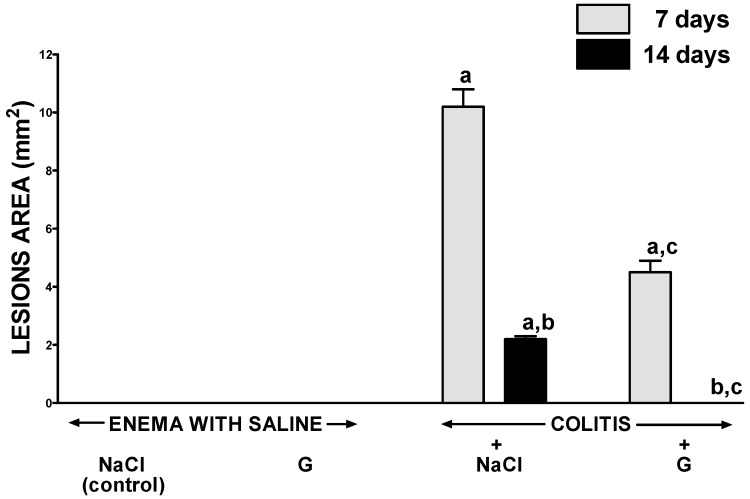
Effect of intraperitoneal administration of saline (NaCl) or ghrelin (G) on the area of colonic mucosa lesions after seven or 14 days from enema with saline or colitis induction. Mean value ± SEM. *n* = 10 animals in each group. ^a^ *p* < 0.05 compared to control at the same time of observation; ^b^ *p* < 0.05 compared to a value observed at the same group after seven days from colitis induction; ^c^ *p* < 0.05 compared to rats with colitis treated with NaCl at the same time of observation.

In rats without the induction of colitis, neither intraperitoneal administration of saline nor ghrelin led to the development of morphological signs of colonic damage (Figure 1). Enema with acetic acid solution induced the development of colitis in all rats tested. In rats treated intaperitoneally with saline, seven days after the induction of colitis by acetic acid enema, the average area of colonic damage was 10.2 ± 0.6 mm^2^; whereas seven days later it was reduced to 2.2 ± 0.1 mm^2^ as a result of spontaneous regeneration (Figure 1). Intraperitoneal administration of ghrelin after induction of colitis accelerated the healing of colonic damage. In rats treated with ghrelin, seven days after rectal administration of acetic acid, the area of colonic damage was reduced by around 60% when compared to lesions observed in the animals without ghrelin treatment. Similar therapeutic effect was observed 14 days after the induction of colitis. Treatment with ghrelin resulted in a full recovery of colonic mucosa and no macroscopic damage have been observed (Figure 1).

**Figure 2:**
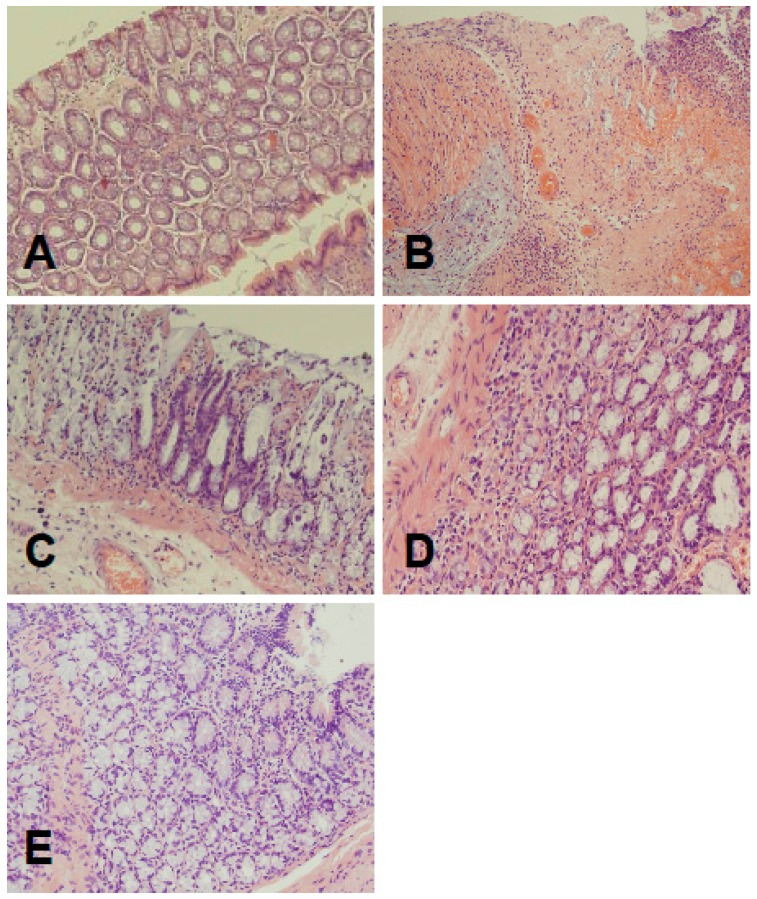
(**A**) Representative microscopic image of colonic mucosa observed in control rats without colitis; (**B**) representative microscopic image of colonic mucosa observed seven days after induction of colitis and i.p. treatment with saline; (**C**) representative microscopic image of colonic mucosa observed seven days after induction of colitis and i.p. treatment with ghrelin; (**D**) representative microscopic image of colonic mucosa observed 14 days after induction of colitis and i.p. treatment with saline; and (**E**) representative microscopic image of colonic mucosa observed 14 days after induction of colitis and i.p. treatment with ghrelin. Hematoxylin-eosin stain. Original magnification 400×.

Microscopic images obtained from control rats without induction of colitis, did not show any damage of the colon (Figure 2, Table 1). In animals treated with saline for seven days after induction of colitis, the presence of large lesions reaching the level of muscular membrane was associated with moderate or heavy inflammatory cell infiltration and the presence of mild or heavy fibrosis (Figure 2, Table 1). Treatment for seven days with ghrelin reduced histological manifestation of colonic damage. Small lesions, reaching submucosa were accompanied with small or moderate inflammatory infiltration and none or mild fibrosis (Figure 2, Table 1). Seven days later, at the 14th day after induction of colitis, histological examination of the colon obtained from saline treated rats showed spontaneous regeneration of colonic wall. Colonic damage was reduced to small lesions reaching submucosa, inflammatory infiltration was reduced to small or moderate. This alteration was associated with the presence of small fibrosis (Figure 2, Table 1). Treatment with ghrelin for 13 days after induction of colitis additionally accelerated spontaneous regeneration of the colon. Histological images showed almost normal colon wall structure apart from the presence of a slight inflammatory infiltration in some cases (Figure 2, Table 1).

### 2.2. DNA Synthesis in Colonic Mucosa

**Figure 3:**
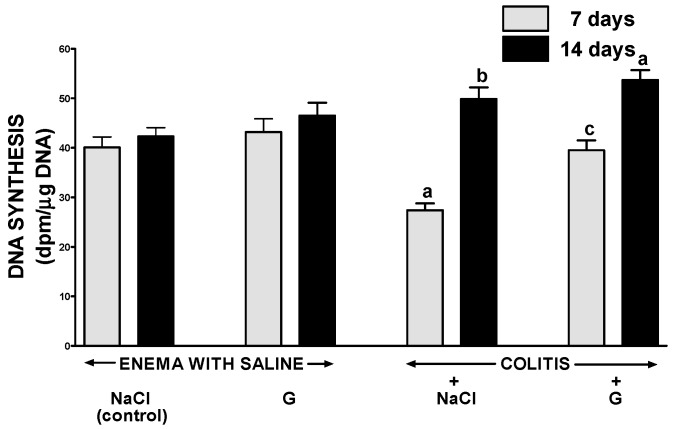
Effect of intraperitoneal administration of saline (NaCl) or ghrelin (G) on the rate of DNA synthesis in colonic mucosa after seven or 14 days from enema with saline or colitis induction. Mean value ± SEM. *n* = 10 animals in each group. ^a^ *p* < 0.05 compared to control at the same time of observation; ^b^ *p* < 0.05 compared to a value observed at the same group after seven days from colitis induction; ^c^ *p* < 0.05 compared to rats with colitis treated with NaCl at the same time of observation.

Administration of ghrelin at the dose used failed to affect significantly DNA synthesis in colonic mucosa in rats without induction of colitis (Figure 3). Induction of colitis by enema with acetic acid reduced mucosal DNA synthesis in the colon. Seven days after induction of colitis, DNA synthesis in the colonic mucosa was significantly reduced by 32%, when compared to a value observed in control group without induction of colitis. Fourteen days after the induction of colitis, DNA synthesis in colonic mucosa of animals treated with saline increased to a value exceeding the level observed in control animals. Administration of ghrelin improved mucosal DNA synthesis in the colon of rats with colitis. Seven days after induction of colitis, this effect was found as a statistically significant and almost complete reversal of the colitis-evoked drop in mucosal DNA synthesis. Seven days later, no marked difference in the rate of mucosal colonic DNA synthesis was observed in both groups of animals with colitis (Figure 3).

### 2.3. Mucosal Blood Flow

**Figure 4:**
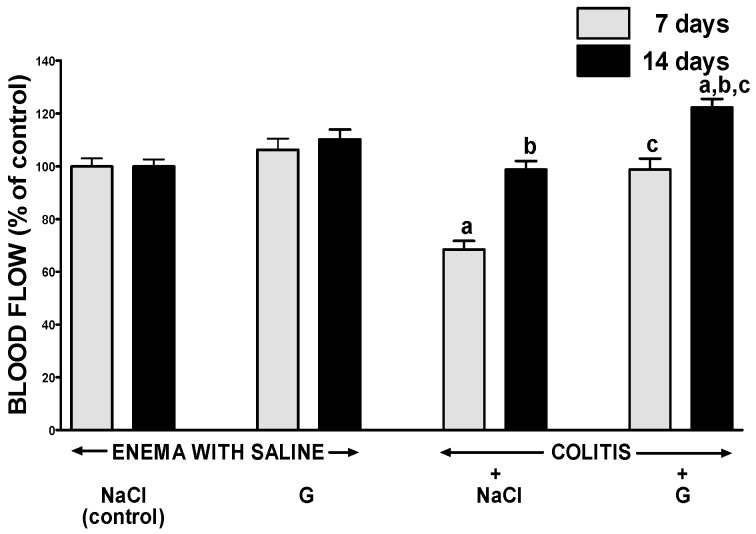
Effect of intraperitoneal administration of saline (NaCl) or ghrelin (G) on blood flow in colonic mucosa after seveb or 14 days from enema with saline or colitis induction. Mean value ± SEM. *n* = 10 animals in each group. ^a^ *p* < 0.05 compared to control at the same time of observation; ^b^ *p* < 0.05 compared to a value observed at the same group after seven days from colitis induction; ^c^ *p* < 0.05 compared to rats with colitis treated with NaCl at the same time of observation.

In groups of animals without induction of colitis, intraperitoneal administration of ghrelin for six or 13 days failed to affect the flow of blood through the mucosa of the large intestine (Figure 4). In rats with colitis, seven days after enema with acetic acid, blood flow through the colonic mucosa was significantly reduced by around 30%, when compared to a value observed in control animals without colitis. Seven days later, mucosal blood flow in the colon of animals with colitis, was spontaneously improved reaching a value similar to that in control animals. In rats with colitis, administration of ghrelin caused a statistically significant improvement of mucosal blood flow in the colon and this effect was observed either seven days or 14 days after induction of colitis (Figure 4).

### 2.4. Mucosal Concentration of Proinflammatory Cytokines and Activity of Myeloperoxidase

**Figure 5:**
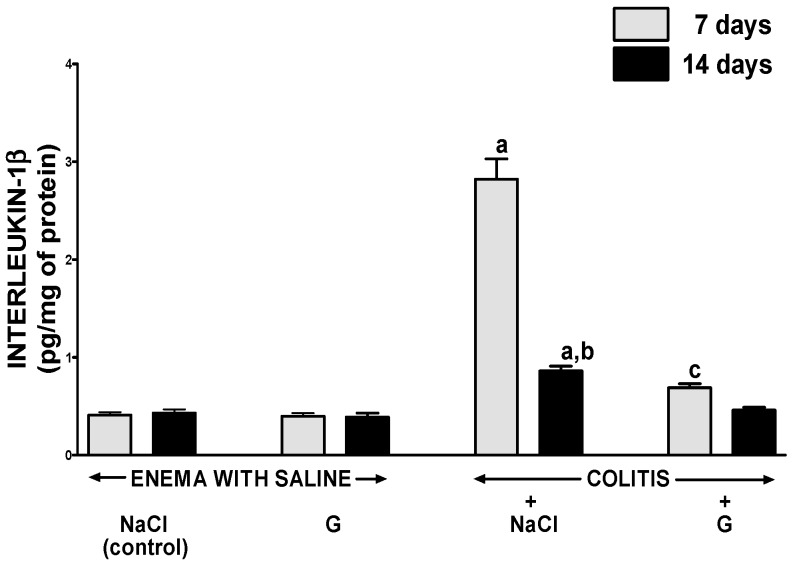
Effect of intraperitoneal administration of saline (NaCl) or ghrelin (G) on concentration of interleukin-1β in colonic mucosa after seven or 14 days from enema with saline or colitis induction. Mean value ± SEM. *n* = 8 animals in each group. ^a^ *p* < 0.05 compared to control at the same time of observation; ^b^ *p* < 0.05 compared to a value observed at the same group after seven days from colitis induction; ^c^ *p* < 0.05 compared to rats with colitis treated with NaCl at the same time of observation.

**Figure 6:**
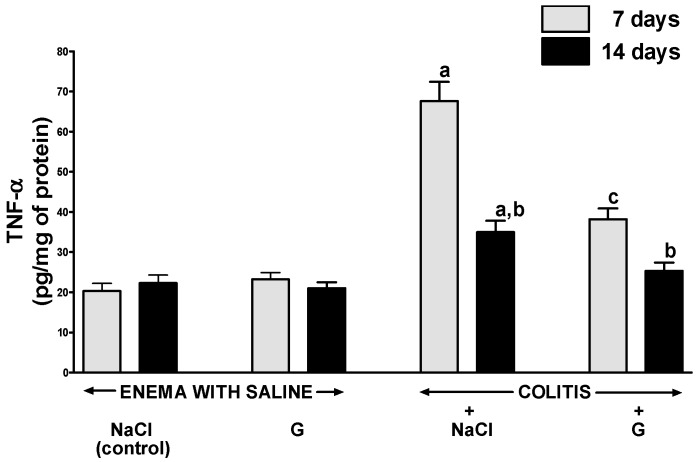
Effect of intraperitoneal administration of saline (NaCl) or ghrelin (G) on concentration of TNF-α in colonic mucosa after seven or 14 days from enema with saline or colitis induction. Mean value ± SEM. *n* = 8 animals in each group. ^a^ *p* < 0.05 compared to control at the same time of observation; ^b^ *p* < 0.05 compared to a value observed at the same group after seven days from colitis induction; ^c^ *p* < 0.05 compared to rats with colitis treated with NaCl at the same time of observation.

In rats without colitis, administration of ghrelin for six or 13 days after rectal enema with saline had no impact on the concentration of interleukin-1β (IL-1β) (Figure 5) or tumor necrosis factor-α (TNF-α) (Figure 6) in the mucosa of the colon. Induction of colitis increased mucosal concentration of IL-1β and TNF-α. Seven days after induction of colitis in rats treated i.p. with saline, almost a seven-fold increase in mucosal concentration of IL-1β in the colon was observed (Figure 5). A value of mucosal concentration of TNF-α was around a three-fold larger than mucosal concentration of TNF-α in the colon of control animals without colitis (Figure 6). Seven days later, mucosal concentration of IL-1β and TNF-α in the colon of rats treated with saline after induction of colitis was still significantly elevated and reached a value around two-fold greater than that observed in saline-treated animals without colitis. In animals with colitis, administration of ghrelin for six days resulted in more than a four-fold reduction in IL-1β in colonic mucosa; whereas concentration of TNF-α was reduced twice (Figure 5 and Figure 6). Fourteen days after induction of colitis, IL-1β and TNF-α concentration in colonic mucosa in rats treated with ghrelin was similar to that observed in animals without induction of colitis (Figure 5 and Figure 6).

**Figure 7:**
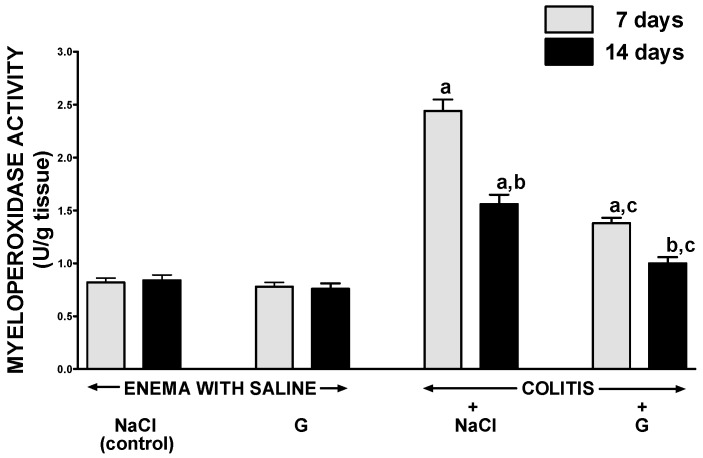
Effect of intraperitoneal administration of saline (NaCl) or ghrelin (G) on activity of myeloperoxidase in colonic mucosa after seven or 14 days from enema with saline or colitis induction. Mean value ± SEM. *n *= 10 animals in each group. ^a^ *p* < 0.05 compared to control at the same time of observation; ^b^ *p* < 0.05 compared to a value observed at the same group after seven days from colitis induction; ^c^ *p* < 0.05 compared to rats with colitis treated with NaCl at the same time of observation.

Intraperitoneal administration of ghrelin for six or 13 days did not affect mucosal myeloperoxidase activity in the colon in rats without colitis induction (Figure 7). Seven days after the induction of colitis, a three-fold increase in myeloperoxidase activity was observed in colonic mucosa as compared to that observed in animals without induction of colitis. Additionally, 14 days after the induction of colitis, myeloperoxidase activity in colonic mucosa was still almost a two-fold higher than in control animals. In rats with colitis, administration of ghrelin resulted in a statistically significant inhibition the colitis-evoked increase in myeloperoxidase activity in colonic mucosa. Seven days after induction of colitis, in rats treated with ghrelin, mucosal activity of myeloperoxidase in the colon was reduced by around 36% when compared to rats with colitis without treatment with ghrelin (Figure 5). Similar effect was observed seven days later.

## 3. Discussion

Our present study has demonstrated that ghrelin administration reduces the inflammation and accelerates healing of acetic acid-induced colitis. Ghrelin administered at a dose of 8 nmol/kg caused a significant decrease in inflammatory lesion area by more than 50% within seven days after the induction of colitis, and complete healing of the colon mucosa seven days later. Earlier research demonstrated that in case of other organs, such as the pancreas [20,22], stomach [15], duodenum [15], and oral cavity [25], ghrelin at doses of eight and 16 nmol/kg/exhibited similar high effectiveness in healing of those organs, therefore, in the presented study, one dose of 8 nmol/kg was chosen. Beneficial effect of ghrelin was accompanied by an improvement of DNA synthesis in the colonic mucosa in rats with colitis in both periods tested. Cell renewal in mucosa is characterised by high dynamics and in the case of the colon, the time of cell renewal of the mucous membrane is from 3–8 days.

Previous studies in the gut have shown that reduction in cell proliferation or excessive apoptosis results in the development of ulcers [38,39]. On the other hand, the increase in cell renewal may cause mucosal hyperplasia, but it also leads to increased protection of the digestive system mucosa against the effects of damaging agents, as well as accelerates the reconstruction of mucosa integrity after damage [40,41,42,43].

In eukaryotes, cell proliferation occurs in the mitotic (M) phase of the cell cycle. The mitotic phase is preceded by interphase, where the cell grows, preparing it for cell division and duplicating its DNA. Interphase consists of three phases: G_1_ (the first gap, also called the growth phase), S (synthesis), and G_2_ (the second gap). DNA synthesis and replication of chromosomes take place only during the S phase of the cell cycle [44]. Therefore, the rate of DNA synthesis, measured by determining the degree of tritium-labelled thymidine built into DNA reflects the vitality and proliferation of cells. Our current study has demonstrated that induction of colitis is accompanied by a decrease in mucosal DNA synthesis below the values observed in the control animals without colitis. Moreover, the decrease in mucosal DNA synthesis in the colon was well-correlated with the size of colonic damage. This observation indicates that reduction in cell proliferation is involved in the pathogenesis of acetic acid-induced colitis in rats.

Spontaneous healing of mucosal damage in the gut leads to mucosal regeneration by migration of surrounding cells from the margin of damage and subsequently by an increase in mucosal cell proliferation [45]. In our present study, treatment with ghrelin for six days significantly improved the rate of mucosal DNA synthesis in the colon in rats with colitis to a value similar to that observed in rats without colitis. Seven days later, in rats with colitis treated with ghrelin, DNA synthesis in colonic mucosa reached a higher value than that in control animals without colitis. This observation suggests that effect of ghrelin on the DNA synthesis in rats with colitis is biphasic. In the first phase of healing, ghrelin improves vitality of mucosal cell; in the second one stimulates cell proliferation in colonic mucosa. A very interesting finding of our study was the observation that treatment with ghrelin at doses used was without significant effect on DNA synthesis in colonic mucosa in rats without induction of colitis. This finding indicates that treatment with ghrelin given at the dose of 8 nmol/kg/dose is safe and does not lead to hyperplasia of colonic mucosa.

Adequate blood flow through the microcirculation plays an essential role in the protection and regeneration of the mucous membrane in gastrointestinal tract [46,47,48]. Research on animals has shown that exposure of gastric mucous membrane to potentially harmful substances results in minimal or no damage, as long as appropriate perfusion of the microcirculation is preserved. On the other hand, the restriction of blood flow through the mucosa results in the formation of large gastric ulcers [46]. Blood flow contributes to protection of mucosa in the gut by delivery of oxygen, bicarbonate, nutrients, hormones, and growth factors, and by removal of carbon dioxide, hydrogen ions, and other toxic agents diffusing from the lumen to gastric or intestinal wall. Local improvement of mucosal blood flow has been shown to reduce mucosal damage and accelerate mucosal healing in the stomach [46], esophagus [49], duodenum [15], and colon [50]. In our present study, we have found that induction of colitis by acetic acid enema leads to initial reduction of mucosal blood flow in the colon, followed by subsequent increase in this parameter during spontaneous healing of colitis. Administration of ghrelin significantly improved mucosal blood flow in the colon leading to faster healing of colitis. These observations indicate that improvement of mucosal blood flow is involved in therapeutic effect of ghrelin in the treatment of acetic acid-induced colitis. Improvement of mucosal blood flow may be a result of increased perfusion of existing vessels or sprouting new blood vessels. Some in vitro and in vivo studies indicate that ghrelin stimulates angiogenesis. Ahluwalia et al. [51] have shown that reduction in ghrelin level in endothelial cells is involved in aging-related impairment of angiogenesis. The promoting role of ghrelin in angiogenesis was also observed by Katare et al. [52]. They found that ghrelin promotes functional angiogenesis in critical limb ischemia through activation of proangiogenic microRNAs and they suggested that ghrelin may be a promising and potent inducer of reparative vascularization in clinical use. Ghrelin has been also found to stimulate angiogenesis in rat model of myocardial infarction [53], and this effect was associated with an anti-apoptotic effect and significant increase in vascular endothelial growth factor (VEGF) expression in the peri-infarct zone. In harmony with these findings is observation that administration of ghrelin ameliorates impaired angiogenesis in ischemic myocardium of diabetic rats with myocardial infarction [54]. This effect has been shown to involve AMPK/eNOS signal pathway by upregulated expression of hypoxia-inducible factor-1α (HIF-1α), VEGF, fetal liver kinase-1 (Flk-1), and fms-like tyrosine kinase-1 (Flt-1).

On the other hand, there are also reports suggesting that ghrelin inhibits angiogenesis. Studies performed on human umbilical vein endothelial cells (HUVECs) have shown that ghrelin inhibits the FGF-2-induced proliferation of these cells and reduces formation of capillary-like structures [55]. Inhibitory effect of ghrelin on the FGF-2-induced angiogenic response was also found in the chick embryo chorioallontoic membrane [55]. Moreover, ghrelin was shown to attenuate angiogenesis induced by low doses of oxidized low-density lipoproteins in human coronary endothelial cells [56]. Additionally, study performed in rats exposed to chronic normobaric hypoxia has indicated that administration of ghrelin reduces the hypoxia-induced angiogenesis and decreases the expression of HIF-1 and VEGF [57].

Another interesting finding of our present study is observation concerning the influence of induction of colitis and administration of ghrelin on mucosal concentration of proinflammatory cytokines, IL-1β, and TNF-α. IL-1β is a central mediator of innate immunity and inflammation. IL-1β is a member of the IL-1 family. This family consists of eleven cytokines and includes seven ligands with agonist activity (IL-1α and IL-1β, IL-18, IL-33, IL-36α, IL-36β, and IL-36γ), three receptor antagonists (IL-1Ra, IL-36Ra, and IL-38), and the anti-inflammatory cytokine (IL-37) [58]. IL-1β plays the most impotent role in the IL-1 family as a proinflammatory factor. IL-1β leads to the development of acute inflammation through activation of local and systemic pro-inflammatory response [59]. IL-1β acts directly, as well as stimulates the release of other pro-inflammatory interleukins in proinflammatory cascade, mainly IL-6, TNF-α, and pro-inflammatory prostaglandins [60]. Numerous experimental and some clinical studies have shown that administration of interleukin-1 receptor antagonist or monoclonal anti-interleukin-1β antibody prevents the increase in serum concentration of IL-6 and TNF-α, and decreases the severity of systemic and local inflammation [60,61,62,63,64]. Our present study has shown that induction of colitis by enema with acetic acid leads to a seven-fold increase in mucosal concentration of IL-1β in the colon at the seventh day after induction of colitis, followed by reduction in mucosal level of IL-1β during subsequent recovery. Similar changes have been observed in the case of TNF-α. Treatment with ghrelin after induction of colitis has inhibited a rise in mucosal IL-1β and TNF-α concentration. Seven days after induction of colitis, a concentration of those pro-inflammatory cytokines in colonic mucosa in rats with colitis treated with ghrelin was only a two-fold higher than in control rats; whereas seven days later, a mucosal concentration of IL-1β and TNF-α was similar to that observed in control rats. These data indicate that ghrelin exhibits strong anti-inflammatory properties and this mechanism is involved in therapeutic effect of ghrelin in acetic acid-induced colitis.

In agreement with our data concerning mucosal concentration of IL-1β are our findings concerning mucosal activity of myeloperoxidase (MPO). MPO is a peroxidase enzyme most abundantly expressed in neutrophil granulocytes. MPO is stored in azurophilic granules and released into extracellular space during inflammatory reactions. MPO catalyses production of hypochlorous acid and tyrosyl radicals; those factors have a strong antibacterial and antiviral effect. However, free radicals, apart from a protective effect against infectious factors, have a damaging influence on body cells, leading to destruction of protein, DNA and lipids [65]. The level of MPO activity adequately reflects the degree of tissue infiltration by neutrophils [65,66]. Our present study has shown that treatment with ghrelin reduces activity of MPO in rats with colitis. These observations confirm that beneficial influence of ghrelin in colitis is, at least in part, related to a decrease in local inflammation. The accuracy of such conclusion is supported by findings that ghrelin inhibits expression of pro-inflammatory cytokines in human monocytes and lymphocyte T cells [67] and decreases the severity of septic shock [68].

There are studies reporting a relationship between elevated MPO activity and the severity of coronary artery disease [69,70]. Moreover, an elevated activity of MPO increases the risk of cardiovascular mortality [70] and plays an important role in the development of the atherosclerotic lesion [71]. These findings taken together with our observation that ghrelin reduces MPO activity in acetic acid-induced colitis may suggest that treatment with ghrelin of colitis may additionally prevent the development of coronary artery disease.

The aim of our present study was to examine whether ghrelin administration affects the course of acetic acid-induced colitis. In the current research, we have not studied the intracellular signaling pathways involved in the therapeutic effects of ghrelin. On the other hand, based on earlier observations of ghrelin’s action on the various organs, it is clear that activation of GHS-R triggers a variety of signaling mechanisms leading to diverse physiological effects. Regulation of effects of GHS-R agonists occurs at the level of receptor transcription, its interaction and internalization. The intracellular signaling pathways of GHS-R stimulation involves phospholipase C (PLC)/inositol (1,4,5) triphosphate (IP_3_) signaling pathway, and the debated protein kinase A (PKA)/cAMP pathway leading to intracellular increase in Ca^2+^ concentration [72]. AMP activated protein kinase (AMPK), mechanistic target of rapamycin (mTOR), MAP kinase (MAPK), and PI3K/AKT signaling pathways may also mediate signal transduction upon activation of GHS-R [72]. The anti-inflammatory effect of ghrelin is probably associated with inhibition of the TGF-β1 and NF-κB pathways [26].

Obestatin is a 23-amino-amino acid peptide derived as ghrelin from the same common for both peptides a 117-amino-acid preproghrelin by post-translational processing [3]. In contrast to ghrelin, obestatin has been postulated to inhibit appetite and its receptor is still unknown. However, previous studies have shown that obestatin affects the development and the course of colitis. Pretreatment with obestatin inhibits the development [47] and accelerates the healing [43] in acetic acid-induced colitis in rats. Similar protective and therapeutic effect of obestatin has been observed in dextran sodium sulfate (DSS)-induced colitis in rats [73]. Those data suggest that both peptides derives from preproghrelin may exhibit beneficial effects on the colonic integrity.

Our current study has shown that administration of exogenous ghrelin accelerates the healing of the acetic acid-induced colitis. This observation is in agreement with previous study showing that ghrelin exhibits therapeutic effect in the course of colitis induced by trinitrobenzene sulfonic acid (TNBS) in rats [30] and mice [32]. Moreover, pretreatment with ghrelin has been shown to inhibit the development of acetic acid-induced colitis in rats [33]. In the case of DSS-induced colitis, the matter of ghrelin’s effects is more complicated. Study performed on mice showed a deleterious effect of endogenous and exogenous ghrelin in DSS-induced colitis [34,74]. In contrast, experiments conducted on rats exhibited the protective and therapeutic effect of ghrelin in this model of colitis [73,75]. These discrepancies indicate that the effect of ghrelin on the course of inflammation may be related to the species of animals used in the research.

## 4. Material and Methods

### 4.1. Animals and Treatment

Studies were performed on 80 male Wistar rats weighing 220–250 g and were conducted following experimental protocol approved by the Local Commission of Ethics for the Care and Use of Laboratory Animals (101/2007, 6 July 2011). During experiments, animals were kept in cages placed in room temperature with a 12 h light-darkness cycle. Animals were fasted with free access to water for 18 h prior to induction of colitis. Later, water and food were available ad libitum.

Animals were randomly divided into four groups: (1) control rats without colitis induction treated intraperitoneally (i.p.) with saline; (2) rats without colitis induction treated i.p. with ghrelin; (3) rats with colitis treated i.p. with saline; and (4) rats with colitis treated i.p. with ghrelin.

In rats anesthetized with ketamine (50 mg/kg i.p., Bioketan, Vetoquinol Biowet, Gorzów Wielkopolski, Poland), colitis was induced by a rectal enema with 1 mL of 3% acetic acid administered thought a polyethylene catheter inserted into the rectum at a depth of 4.5 cm. Rats without induction of colitis obtained a rectal enema with aqueous saline solution administered at the same manner as the solution of acetic acid in animals with the induction of colitis.

Starting 24 h after a rectal enema with saline or acetic acid, rats were treated with saline (groups 1 and 3) or ghrelin (groups 2 and 4) administered i.p. twice a day. Rat ghrelin (Yanaihara Institute, Shizuoka, Japan) was given at the dose of 8 nmol/kg/dose. This dose was chosen because previous studies have shown that ghrelin given at the dose 8 nmol/kg/dose exhibits strong and repeatable therapeutic effect in the healing of gastric, duodenal and oral ulcers [15,25]. Rats from each experimental group were randomly divided into two subgroups. In the first subgroup, the healing rate of the colon was evaluated seven days after acetic acid enema. In the second subgroup evaluation was performed after next seven days. Each subgroup consisted of 10 animals.

### 4.2. Measurement of Colonic Blood Flow and Colonic Damage

At the end of experiments animals were anesthetized again with ketamine. After opening the abdominal cavity and exposure of the colon, the rate of colonic blood flow was measured using a laser Doppler flowmeter (PeriFlux 4001 Master monitor, Perimed AB, Järfälla, Sweden), in accordance with the methodology described before [76]. The blood flow measurement was performed each time in five parts of the descending and sigmoid colon and a main value of five records was expressed as the percentage of value obtained in animals from the control group. After measurement of the colonic blood flow, the area of mucosal damage was measured, using a computerized planimeter (Morphomat, Carl Zeiss, Berlin, Germany), in accordance to the method described earlier [17].

### 4.3. Biochemical Analysis

Biopsy samples of colonic mucosa or colonic wall were taken for and determination of mucosal DNA synthesis (an index of mucosal cell vitality and proliferation), interleukin-1β (IL-1β) and tumor necrosis factor-α (TNF-α) concentration, myeloperoxidase activity, and histological examination. DNA synthesis in the colonic mucosa was determined by radioisotope method by measuring titrated thymidine incorporation into DNA as previously described [77]. Briefly, samples of mucosa were crushed with scissors, and then incubated at 37 °C for 45 min in 2 mL of nutrient solution containing thymidine labeled with tritium (8 μCi/mL) ([6-^3^H]thymidine, 20–30 Ci/mmol; Institute for Research, Production and Application of Radioisotopes, Prague, Czech Republic). Incorporation of [^3^H]-thymidine into DNA was determine by counting 0.5 mL DNA-containing supernatant in liquid scintillation system. DNA synthesis was expressed as disintegration per minute [^3^H]-thymidine per microgram DNA (dpm/μg DNA).

Samples of the colonic mucosa for assessing the concentration of IL-1β and TNF-α were homogenized in phosphate buffer at 4 °C. Then the homogenate was centrifuged and the concentration of IL-1β and TNF-α was determined in the supernatant using the BioSource Cytoscreen rat IL-1β kit (BioSource International, Camarillo, CA, USA) or rat TNF-α ELISA Kit (Koma Biotech, Seoul, Korea), respectively. The concentration of IL-1β and TNF-α in the colonic mucosa was expressed in pg/mg of proteins.

Biopsy samples for measurement of mucosal myeloperoxidase activity were frozen in liquid nitrogen and then, until the time of the determination, stored at −60 °C. Determination of myeloperoxidase activity was performed using a modification of the method described by Bradley [78]. Results obtained in units per gram of tissue, were finally expressed as a percentage of the value observed in the control group.

### 4.4. Histological Examination of the Colon

Samples of the colon were fixed in 10% buffered formaldehyde and embedded in paraffin. Paraffin sections were stained with hematoxylin and eosin, and examined by the pathologist uninformed about treatment given. The histological grading of colonic damage such as ulceration, inflammation, depth of the lesion and fibrosis, was determined using a scale of Vilaseca [79] as described in detail previously [33].

### 4.5. Statistical Analysis

Results were presented as mean value ± standard error (SEM). Statistical evaluation was performed by analysis of variance followed by Tukey’s multiple comparison test using GraphPad Prism (GraphPad Software, San Diego, CA, USA). Differences were considered statistically significant when *p* was less than 0.05.

## 5. Conclusions

In conclusion we can say that administration of ghrelin accelerates the healing of the acetic acid-induced colitis and this effect involves the ghrelin-evoked reduction in inflammation associated with improvement of mucosal blood flow and an increase in mucosal cell proliferation. This observation, taken together with previous findings that ghrelin exhibits a therapeutic effect in trinitrobenzene sulfonic acid- and dextran sodium sulfate-induced colitis, suggest that the therapeutic effect of ghrelin in the colon is universal and independent of the primary cause of colitis.

## Figures and Tables

**Table 1:** Effect of intraperitoneal administration of saline (NaCl) or ghrelin on morphological signs of colonic damage observed seven or 14 days after enema with saline or induction of colitis.

	Morphological Changes			
Groups	Grading of Colonic Damage (0–2)	Inflammatory Infiltration (0–3)	Depth of Damage (0–3)	Fibrosis (0–3)
Seven days				
Enema With Saline + Nacl (Control)	0	0	0	0
Enema With Saline + Ghrelin	0	0	0	0
Colitis + Nacl	2	2–3	2	1–2
Colitis + Ghrelin	1	1–2	1	0–1
14 days				
Enema With Saline + Nacl (Control)	0	0	0	0
Enema With Saline + Ghrelin	0	0	0	0
Colitis + Nacl	1	1–2	1	1
Coltis + Ghrelin	0	0–1	0	0

Numbers represent the predominant histological grading in each group.
